# Effect of the Secondary Symbiont *Hamiltonella defensa* on Fitness and Relative Abundance of *Buchnera aphidicola* of Wheat Aphid, *Sitobion miscanthi*

**DOI:** 10.3389/fmicb.2018.00582

**Published:** 2018-03-29

**Authors:** Li Qian, Fan Jia, Sun Jingxuan, Wang Manqun, Chen Julian

**Affiliations:** ^1^The State Key Laboratory for Biology of Plant Diseases and Insect Pests, Institute of Plant Protection, Chinese Academy of Agricultural Sciences, Beijing, China; ^2^College of Plant Science and Technology, Huazhong Agricultural University, Wuhan, China

**Keywords:** *Sitobion miscanthi*, symbionts, *Hamiltonella defensa*, insect fitness, *Buchnera aphidicola*, relative abundance

## Abstract

Bacterial symbionts associated with insects are often involved in host development and ecological fitness. In aphids, the role of these symbionts is variable and not fully understood across different host species. Here, we investigated the symbiont diversity of the grain aphid, *Sitobion miscanthi* (Takahashi), from 17 different geographical areas. Of these, two strains with the same symbiont profile, except for the presence of *Hamiltonella defensa*, were selected using PCR. The *Hamiltonella*-infected strain, YX, was collected from a Yuxi wheat field in Yunnan Province, China. The *Hamiltonella*-free strain, DZ, was collected from a Dezhou wheat field in Shandong Province, China. Using artificial infection with *H. defensa* and antibiotic treatment, a *Hamiltonella*-re-infected strain (DZ-H) and *Hamiltonella*-significantly decreased strain (DZ-HT) were established and compared to the *Hamiltonella*-free DZ strain in terms of ecological fitness. Infection with the DZ-H strain increased the fitness of *S. miscanthi*, which led to increases in adult weight, percent of wingless individuals, and number of offspring. Meanwhile, decreased abundance of *H. defensa* (DZ-HT strain) resulted in a lower adult weight and wingless aphid rate compared to the DZ-H strain. However, the indices of longevity in both the DZ-H and DZ-HT strains decreased slightly, but were not significantly different, compared to the DZ strain. Furthermore, quantitative PCR showed that the relative abundance of the primary symbiont *Buchnera aphidicola* in the DZ-H strain was significantly higher than in the DZ strain in all but the first developmental stage. These results indicate that *H. defensa* may indirectly improve the fitness of *S. miscanthi* by stimulating the proliferation of *B. aphidicola*.

## Introduction

Many insects harbor various types of maternally inherited microbial symbionts (Baumann, 2005). A classic model for heritable symbiosis is the association of aphid and its primary (P-) symbiont *Buchnera aphidicola*, many aphid species first colonized an aphid ancestor 150 million years ago and which persists in almost all of the 5,000-aphid species (Baumann et al., 1995; Clark et al., 2000). *B. aphidicola* plays a prominent role in insect nutritional ecology by providing essential nutrients that are not obtained in sufficient amounts from a restricted diet of plant phloem (Baumann et al., 1995). Previous studies have shown that in addition to *B. aphidicola*, a wide range of heritable secondary (S-) symbionts are found in aphids (Buchner, 1965; Oliver et al., 2006; Vorburger and Gouskov, 2011). In contrast to *B. aphidicola*, which is strictly housed in bacteriocytes, S-symbionts are scattered in sheath cells and aphid hemolymph. They are not thought to be essential to host survival (Douglas, 1989). However, recent studies have revealed the important role of S-symbionts. These symbionts may confer conditional adaptive advantages to their host, such as protecting the insect host against pathogens and natural enemies (Piel, 2002; Oliver et al., 2003, 2005; Guay et al., 2009), ameliorating the detrimental effects of heat (Douglas, 1989, 1998; Ohtaka and Ishikawa, 1991; Montllor et al., 2002), enhancing insecticide resistance, aiding in adaptation to the plant (Leonardo and Muiru, 2003; Tsuchida et al., 2004; Guidolin and Cônsoli, 2017), and mediating insect host metabolism and biosynthesis (Akman and Douglas, 2009; Benoit et al., 2017). With the continuous development of molecular biology and omics technologies, greater knowledge of the evolutionary relationships between symbionts and host insects has been revealed (Degnan et al., 2009, 2010; Manzanomarín and Latorre, 2014; Cassone et al., 2015).

There are many S-symbionts that have been identified in aphids, including *Serratia symbiotica, Hamiltonella defensa, Regiella insecticola, Rickettsia, Wolbachia, Spiroplasma, Arsenophonus* (Tsuchida et al., 2002; Oliver et al., 2010), and SMLS (*Sitobion miscanthi* L type symbiont) (Li et al., 2016). *H. defensa* is a well-studied S-symbiont of insects and a gammaproteobacterium informally known as the pea aphid *Bemisia-*like symbiont or T-type (Darby et al., 2001), which is widely, but not universally, distributed in natural populations of aphid, especially in the grain aphid, *Sitobion miscanthi* Takahashi.

*Sitobion miscanthi*, a dominant grain aphid species in China, is a major wheat pest that frequently harbors S-symbionts. *H. defensa* has been comprehensively studied in *Acyrthosiphon pisum* (Nyabuga et al., 2010; Łukasik et al., 2013; Mclean and Godfray, 2015), but most of these studies focused on the role of *H. defensa* in helping the insect during pathogen or parasite invasion via the presence of a lysogenic lambdoid bacteriophage designated APSE (Moran et al., 2005a; Degnan and Moran, 2008). *Hamiltonella*-induced tolerance to high temperature has also been reported (Russell and Moran, 2006). Sporadic reports have noted the positive effect of *H. defensa* on aphid fecundity (Łukasik et al., 2013), but there is still relatively little information available on the ecological adaptation of *H. defensa* to the aphid host, especially in the wheat aphid *S. miscanthi*.

In general, artificial infection and antibiotic elimination studies offer a powerful opportunity to study the influence of symbionts on the host. Thus, in the case of presented study system, *H. defensa* was transferred by microinjection and selectively reduced by antibiotic treatment to provide a host line for the experimental assays. Different geographical populations of aphids naturally harbor various symbionts and provide a source of symbiont diversity for aphid-symbiont interaction research (Tsuchida et al., 2002; Zhao et al., 2016). Aphids with a single strain of S-symbiont are scarce in nature and hence the collection and identification of secondary symbionts in different geographical isofemale strains of *S. miscanthi* were the foundation for the target symbiont screening.

This study was conducted to assess the diversity of S-symbionts in 17 different geographical populations of *S. miscanthi* in China, from which the *Hamiltonella*-infected and *Hamiltonella*-free strains were obtained. A new *Hamiltonella*-re-infected strain and a *Hamiltonella*-significantly decreased strain with the identical genetic backgrounds was generated to evaluate the impact of *Hamiltonella* on host ecological fitness. Furthermore, the relative abundance of the *B. aphidicola* in each development stage was measured in *Hamiltonella*-free and *Hamiltonella*-re-infected strains. Our study clearly identifies the diversity of S-symbionts in *S. miscanthi* and reveals the effect of *H. defensa* on host aphid ecology adaptation and the correlation with *B. aphidicola*.

## Materials and methods

### Aphid rearing

**Figure 1:**
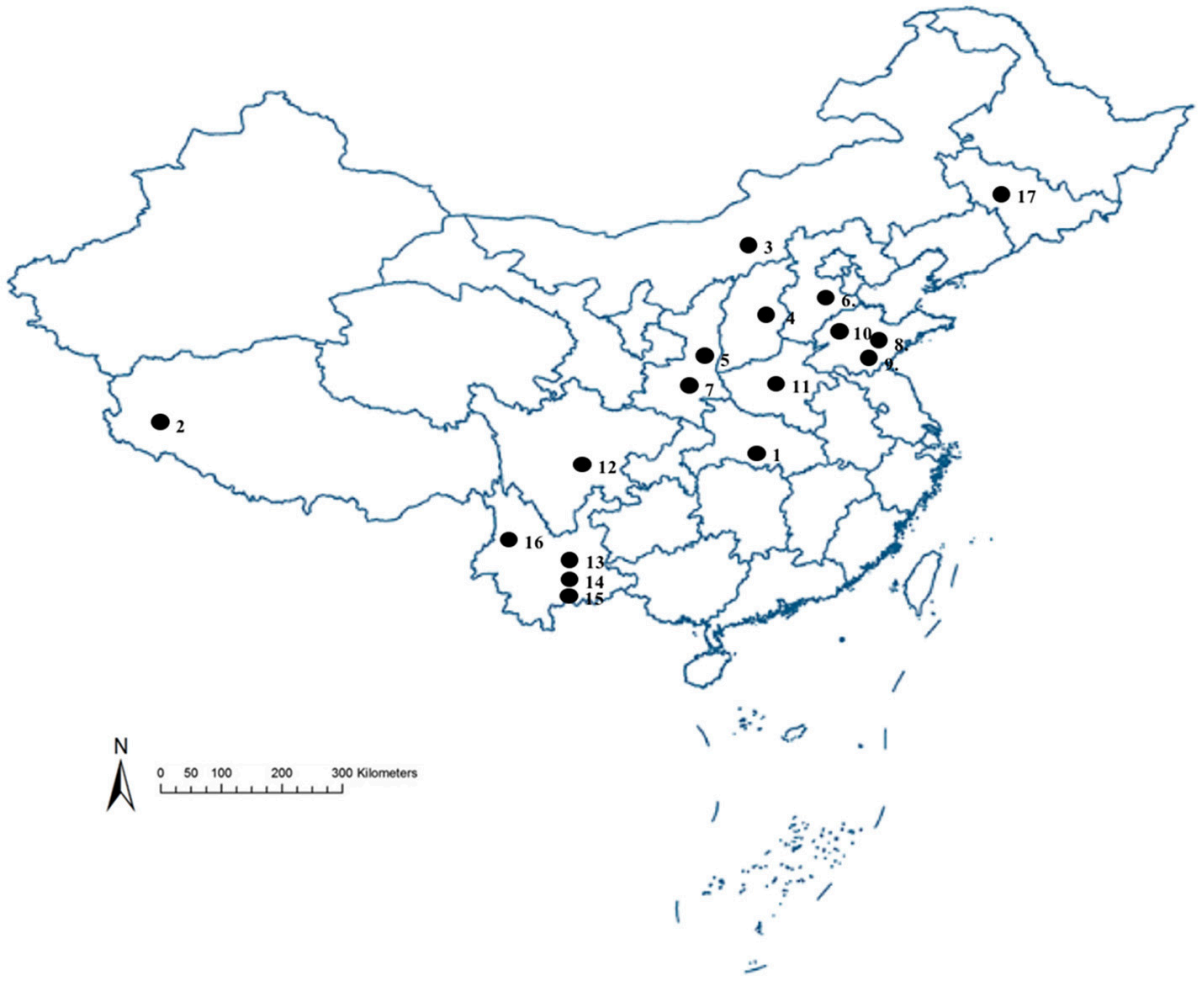
Sampling sites for 17 geographical populations collection to investigate the diversity of secondary symbionts in different geographical *S. miscanthi* strains in China Numbers on the map correspond to locality numbers in Table 1.

The strains of *S. miscanthi* used in this study are shown in Figure 1 and Table 1. These samples were collected from 17 locations covering a wide range of the main wheat-producing areas in mainland China. All of these isofemale strains were established from different collections, feeding separately on aphid-susceptible wheat seedlings (*Triticum aestivum* L) in the culture room at 20 ± 1°C with a 75% relative humidity and a light: dark photoperiod of 16:8 h. After 10 generations, the aphids were used for the following experiments.

**Table 1:** Locality information and diversity of secondary symbionts infection in the different geographic isofemale strains of *S. miscanthi*.

**No**.	**Locality ID**	**Locality**	**Collected time**	***S. symbiotica***	***H. defensa***	***R. insecticola***	***Rickettsia***	***Wolbachia***	***Arsenophonus***	***Spiroplasma***
1	WH	Hubei, Wuhan	2016/3/15			+	+			+
2	LS	Tibet, Lasa	2015/9/1	+		+	+			+
3	HHT	Inner Mongolia, Hohhot	2015/9/1				+			+
4	TY	Shanxi, Taiyuan	2015/5/14			+				+
5	LF	Shanxi, Linfen	2015/6/24			+				+
6	LFang	Hebei, Langfang	2016/3/4			+	+			+
7	WN	Shangxi, Weinan	2016/4/25			+	+			+
8	JN	Shandong, Jinan	2016/4/23			+				+
9	TA	Shandong, Taian	2016/4/22			+				+
10	DZ	Shandong, Dezhou	2016/5/7			+				+
11	XX	Henan, Xinxiang	2016/3/10			+	+			+
12	CD	Sichuan, Chengdu	2015/6/24			+	+			+
13	KM	Yunan, Kunming	2016/4/15		+	+				+
14	YX	Yunan, Yuxi	2016/3/29		+	+				+
15	HH	Yunan, Honghe	2016/3/29		+	+				+
16	DL	Yunan, Dali	2016/4/15		+	+				+
17	CC	Jilin, Changchun	2015/9/30			+				+

### Aphid DNA extraction, sequencing, and secondary symbiont identification

Total aphid DNA was extracted from a single adult aphid with the DNeasy Blood & Tissue Kit (Qiagen, Valencia, CA) following the manufacturer's protocol. DNA was extracted from a total of 20 replicates for each geographical strain. DNA quality was evaluated using a NanoDrop-1000 spectrophotometer (Nano-Drop Technologies, Wilmington, DE). These samples were then screened for the seven known S-symbionts of pea aphids, *S. symbiotica, H. defensa, R. insecticola, Rickettsia, Wolbachia, Spiroplasma*, and *Arsenophonus* (Tsuchida et al., 2002; Oliver et al., 2010) with PCR using the specific symbiont primers listed in Table 2. Cycling conditions were 94°C for 4 min, followed by 35 cycles at 94°C for 30 s, 60°C for 45 s, 72°C for 1 min, and 4°C for the final elongation. The reaction products were analyzed with a model 3500 ABI PRISM DNA sequencer (Perkin-Elmer, New York, NY). The nucleotide sequence of the *H. defensa 16S rRNA* partial gene from *S. miscanthi* described in this paper has been deposited in GenBank under accession number MG721025.

**Table 2:** Secondary symbiont specific primers used in this study.

**Application**	**Target symbiont**	**Primer name**	**Sequence (5′-3′)**	**Product (kb)**	**References**
PCR	*S. symbiotica*	16SA1 PASScmp	AGAGTTTGATCMTGGCTCAG	0.48	Fukatsu et al., 2000
			GCAATGTCTTATTAACACAT		
	*R. insecticola*	U99F 16SB4	ATCGGGGAGTAGCTTGCTAC	0.2	Tsuchida et al., 2002
			CTAGAGATCGTCGCCTAGGTA		
	*H. defensa*	*Hami*F 16SB1	AGCACAGTTTACTGAGTTCA	1.66	Darby et al., 2001
			TACGGYTACCTTGTTACGACTT		
	*Rickettsia*	16SA1 Risk16SR	AGAGTTTGATCMTGGCTCAG CATCCATCAGCGATAAATCTTTC	0.2	Fukatsu and Nikoh, 1998
	*Spiroplasma*	16SA1 TKSSspR	AGAGTTTGATCMTGGCTCAG	0.51	Fukatsu et al., 2000
			TAGCCGTGGCTTTCTGGTAA		
qPCR	*B. aphidicola*	*Buch*-q-F *Buch*-q-R	GGGAACTCAGAGGAGACTGC	0.18	In this study
			TGAGGTTTGCTTGTCTTTGC		
	*H. defensa*	*Hami*-q-F *Hami*-q-R	TGAACAATGTCCCAACTGCT	0.16	In this study
			CGCCTCATCTTTCCTGGTAT		
	*R. insecticola*	*Reg*-q-F *Reg*-q-R	GGTAATACGGAGGGTGCGAG	0.20	In this study
			ACTCTAGCCAGCCAGTCTCA		
	*Spiroplasma*	Spir-q-F Spir-q-R	TGGGATAACTCCGGGGAAACC	0.18	In this study
			TGGTAAACCGGTACCCTTCC		
	ß*-actin*	ß*-actin* F ß*-actin* R	CGTTACCAACTGGGACGATATG	0.16	In this study
			GCGTTCAATGGAGCTTCTGTTA		

### Artificial infection of *H. defensa* by hemolymph injection

The injection of *H. defensa* followed a previously described method (Koga et al., 2003). Briefly, 0.5 μl of hemolymph was extracted from the YX strain and diluted with 0.5 μl of 0.01 M PBS. A 1 μl dilution was injected into the body of third-instar nymphs of the DZ strains. We detected the infection of *H. defensa* in the newborn nymphs from the offspring of the treated aphids 2 weeks after injection. Presence of *H. defensa* was confirmed again after three generations and before the start of the fitness experiment.

### *H. defensa* elimination by antibiotic treatment

To verify the fitness effect of *H. defensa* on *S. miscanthi*, a *Hamiltonella*-significantly decreased strain was established via antibiotic treatment. With a litter modified by the previous treatment, a mixture of the antibiotics ampicillin and gentamycin (Dykstra et al., 2014), each at 100 μg/ml, was added to a 20% sucrose solution as the artificial diet, with the control treatment containing antibiotic-free sucrose solution, for two days. The aphids that fed on the antibiotic artificial solution are referred to as *Hamiltonella*-significantly decreased aphids. Following antibiotic treatment, all of the adult aphids were placed on wheat seedlings to produce new nymphs. After rearing for 10 generations, the offspring of aphids were screened for abundance of *B. aphidicola* and S-symbionts by quantitative PCR. The feeding apparatus was prepared according to Chen et al. (2000) with 250 μl of solution diet sandwiched between two layers of parafilm membrane and stretched to a glass tube of 21-mm diameter under sterile conditions.

### Fitness measurement

The nymphs of DZ (collected from Dezhou wheat filed), DZ-H (a new strain created by DZ strain with *H. defensa* artificial infection) and DZ-HT (a new strain created by DZ-H strain with antibiotic treatment) were collected over 24 h from adult aphids. Thirty nymphs from strains were selected at random and individually placed in petri dishes containing wheat seedlings whose roots were inserted into water in 1.5 ml tubes and kept at 20°C under a long-day (16 h) light cycle. These nymphs were allowed to develop to adults in the petri dishes, and the fitness indices wingless aphid rate, total number of offspring and longevity were checked daily until all nymphs completed their whole lifecycle. We measured the weight of 30 newly emerged adults collected from each strain and performed 6 replications. To eliminate any adverse effects of the injection or antibiotics on aphids, the DZ-H and DZ-HT strains were reared for 10 generations before performing the fitness experiment.

### Quantitative PCR

DNA was extracted from different developmental stages of aphids of the DZ-H and DZ-HT strains within 24 h. To examine whether *H. defensa* infection in aphids influences the relative abundance of the primary symbiont *B. aphidicola*, symbiont relative abundance was quantified by the SYBR Green ROX mix (Takara, Dalian, China) and ABI Prism 7,500 Sequence Detection System (Thermo Fisher Scientific, Waltham, MA, USA). *B. aphidicola, H. defensa, R. insecticola*, and *Spiroplasma* were quantified in terms of *16S rRNA* gene using the primers in the Table 2. The *S. miscanthi* actin gene with primers ß*-actin* was used as an internal standard for data normalization. Three replicates were performed. The amplification efficiency amplified with primers were 103.4, 99.1, 95.5, 97.2, and 101.9% for *B. aphidicola, H. defensa, R. insecticola*, and *Spiroplasma* and actin, respectively.

Quantitative PCR (QPCR) was carried out in 20-μl volumes containing 10 μl of SYBR Green PCR Mix (Takara Bio, Shiga, Japan), 0.4 μl of 50x ROX Dye II, 1 μl of each primer, and 1 μl of DNA. Cycling conditions were 95°C for 15 min, then 40 cycles of 15 s at 95°C, 30 s at the annealing temperature at 60°C, and 30 s at 72°C. The relative abundance of aphid *B. aphidicola* was normalized to the aphid housekeeping gene ß*-actin* and calculated using the comparative C_t_ method 2^−ΔΔ*ct*^method (Vandesompele et al., 2002).

### Statistical analyses

The treatment effects were analyzed by nonparametric tests. Kruskal-Wallis was used for multiple comparisons and Mann-Whitney was used for two sample comparisons at α = 0.05. All of the data analyses were performed using the IBM SPSS statistics Version 21 (ver. 21, SPSS Inc., Chicago, IL, USA) software.

## Results

### Diversity of secondary symbiont infection in different geographical isofemale strains of *S. miscanthi*

The diversity of S-symbiont infections of *S. miscanthi* was investigated from 17 isofemale strains by specific PCR detection. *S. symbiotica, H. defensa, R. insecticola, Rickettsia, Arsenophonus*, and *Spiroplasma* exhibited partial infections in different geographical strains. However, *Wolbachia* was not detected from any of the Chinese strains of *S. miscanthi* (Table 1). When an S-symbiont was detected in a population, infection rate was close to 100%. In these isofemale strains, no single S-symbiont infected strain was detected.

### *H. defensa* artificial reinfection and antibiotic elimination

**Figure 2:**
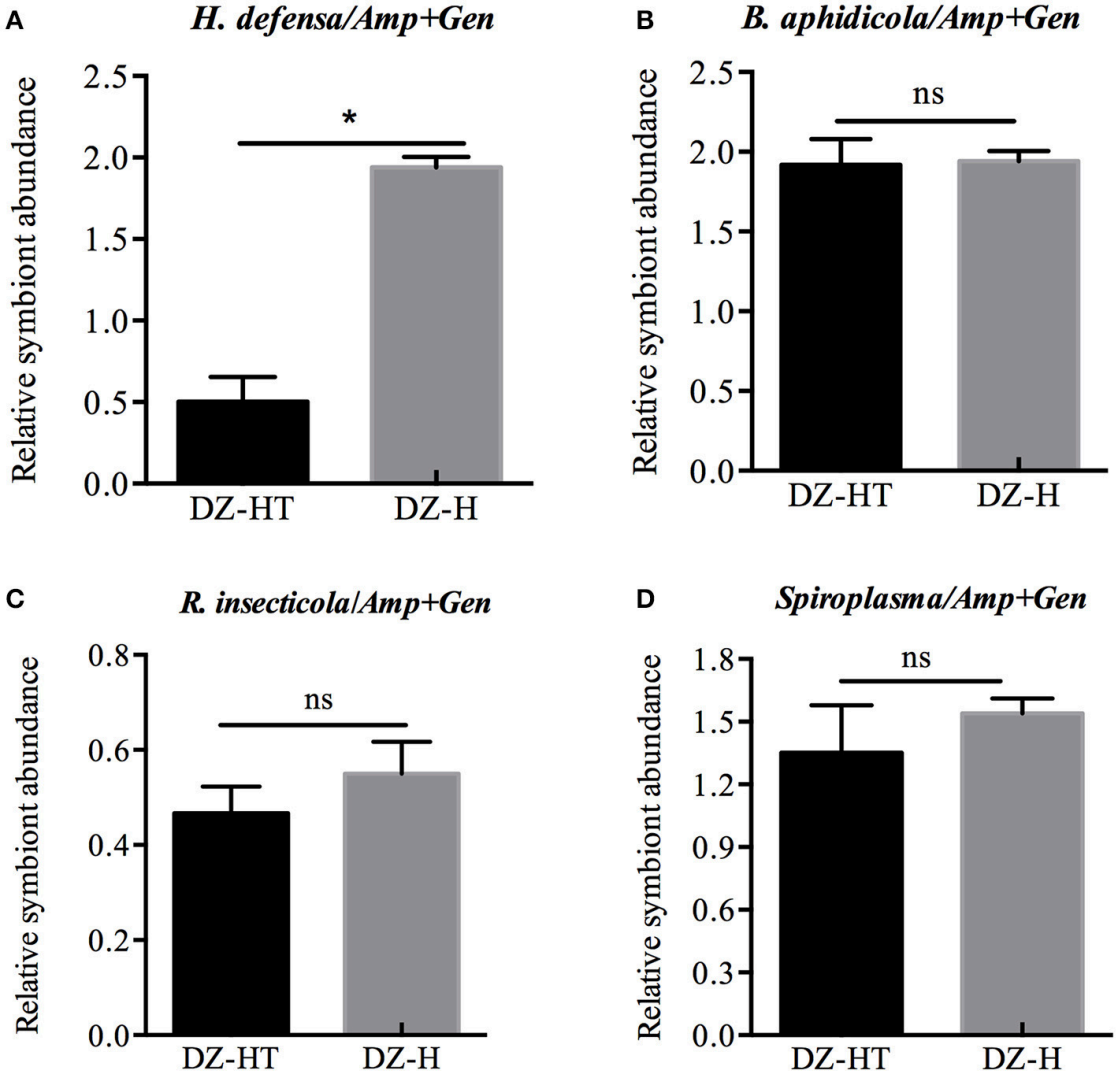
Relative symbiont abundance change with antibiotic treatment designed to specifically eliminate *H. defensa* QPCR analysis of DNA extracted from antibiotic-treated aphids and controls. **(A)** The abundance of *H. defensa* significantly declined in DZ-HT strains treated with antibiotics compared to the control DZ-H strains (*P* < 0.05). Antibiotic treatment had no effect on the abundance of *B. aphidicola, R. insecticola*, and *Spiroplasma* **(B–D)**. The asterisk indicates significant differences based on the Mann-Whitney *U*-test for two sample comparison at *P* < 0.05 and ns indicates no significant difference.

Because no *H. defensa* single infected strains were found in different isofemale strains and given the negative impact of using multiple antibiotics to remove different symbionts, we performed the following experiments using the two strains with the same symbionts background, except *H. defensa*. Twenty third-instar nymphs of the *Hamiltonella*-free DZ strain of *S. miscanthi* were injected with hemolymph obtained from the YX strain of *S. miscanthi*, and the *Hamiltonella*-re-infected strain DZ-H with the identical background of DZ strain was stably established. To evaluate whether *H. defensa* had been transferred into the aphids, DNA samples obtained from sets of 10 first-instar nymphs were subjected to PCR detection based on the *H. defensa 16S rRNA* gene with two replications (data not shown). The sequence of *H. defensa* in our study, 1380-bp in length, exhibited 99.8% similarity to the sequence of *H. defensa* from *A. pisum*. After antibiotic treatment, DZ-HT strains were reared for 10 generations and, then, we estimated the relative abundance of symbionts in DZ-HT strains to ensure that the antibiotic did not affect the other symbionts, except *H. defensa*. Compared to the DZ-H strain, the abundance of *H. defensa* diminished by approximately 4.15-fold (*P* < 0.05; Figure 2A), while *B. aphidicola, R. insecticola*, and *Spiroplasma* showed almost no change (Figures 2B–D). These results showed that a moderate concentration of mixed antibiotics could specifically decrease targeted symbionts without affecting the other symbionts.

### Role of *H. defensa* on host aphid fitness

**Figure 3:**
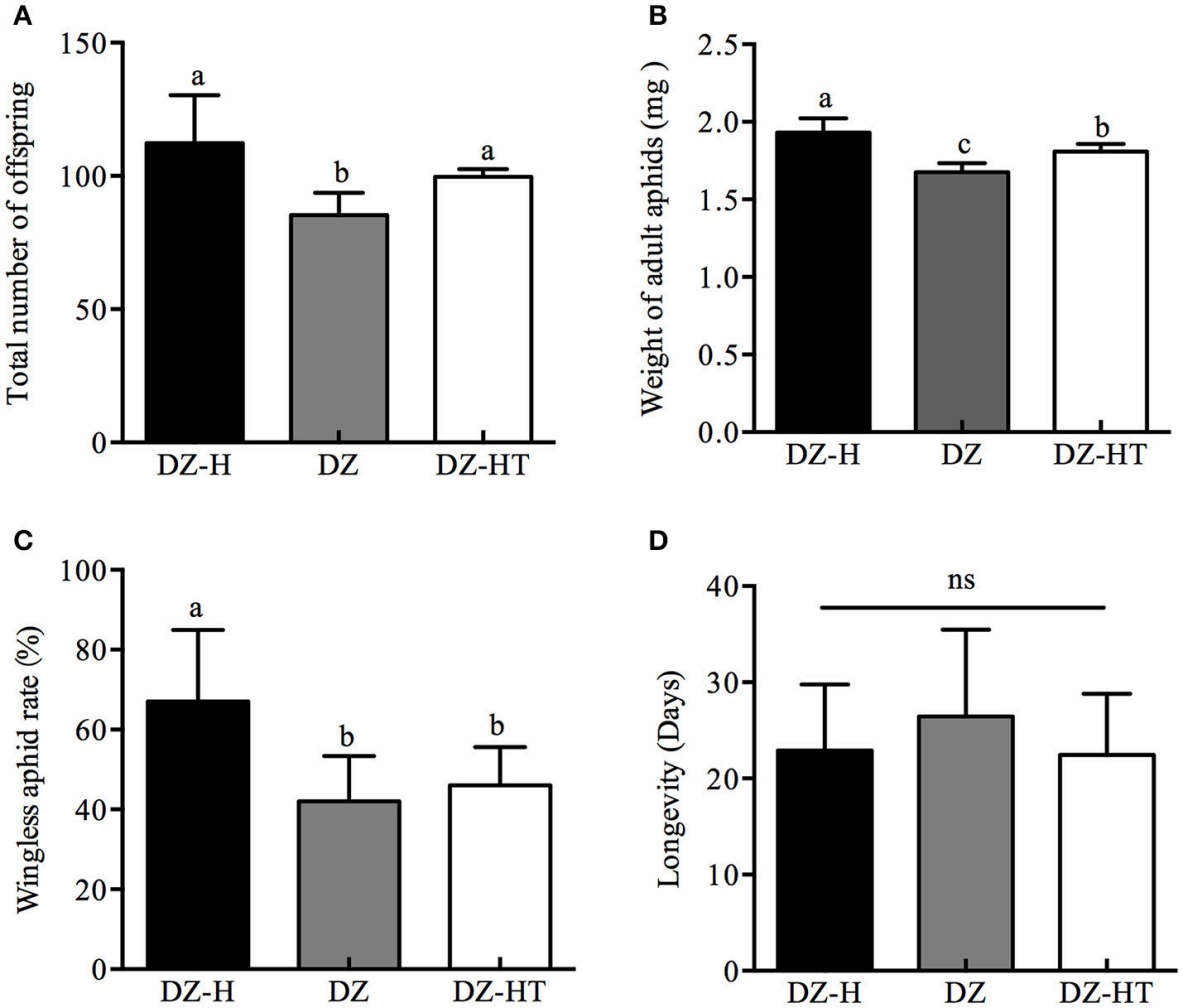
Fitness indices of *Hamiltonella*-infected, *Hamiltonella*-free, and *Hamiltonella*-decreased *S. miscanthi* strains. **(A)** Total number of offspring. **(B)** Weight of adult aphids. **(C)** Percentage of wingless aphids. **(D)** Longevity. Bar represents standard errors of means and different letters above the bars indicate significant differences based on Kruskal-Wallis for multiple comparisons at *P* < 0.05, while ns indicates no significant difference.

Aphid demographic parameters, including adult aphid weight, percentage of wingless aphids, total number of offspring, and longevity were compared among the DZ-H (*Hamiltonella*-re-infected), DZ (*Hamiltonella*-free), and DZ-HT strains (*Hamiltonella*-decreased). The DZ-H strain had a significant positive effect on partial fitness indices compared to the DZ and DZ-HT strains (Figure 3). The total number of offspring per five adults of the DZ-H strain was 112.3, which was significantly higher than the DZ strain (89.3) (*t* = 3.326, *P* < 0.05), but it was not significantly different from the DZ-HT strain (Figure 3A). For the DZ-H strain, the adult aphid weight was 1.93 g, which was significantly higher than the DZ (1.68 g) (*t* = 5.677, *P* < 0.05) and DZ-HT strains (1.808 g) (*t* = 2.814, *P* < 0.05) (Figure 3B). The wingless aphid rate in the DZ-H strain was 67%, which was significantly higher than in the DZ (42%) (*t* = 3.727, *P* < 0.05) and DZ-HT strains (46%) (*t* = 3.316, *P* < 0.05) (Figure 3C). However, the longevity of the DZ-H strain was 22.9 days, which was not significantly different from the DZ (26.4 days) (*t* = 1.705, *P* = 0.09) and DZ-HT strains (22.4 d) (*t* = 0.278, *P* = 0.79) (Figure 3D).

### Relative abundance difference in *B. aphidicola* between DZ-H and DZ strains

**Figure 4:**
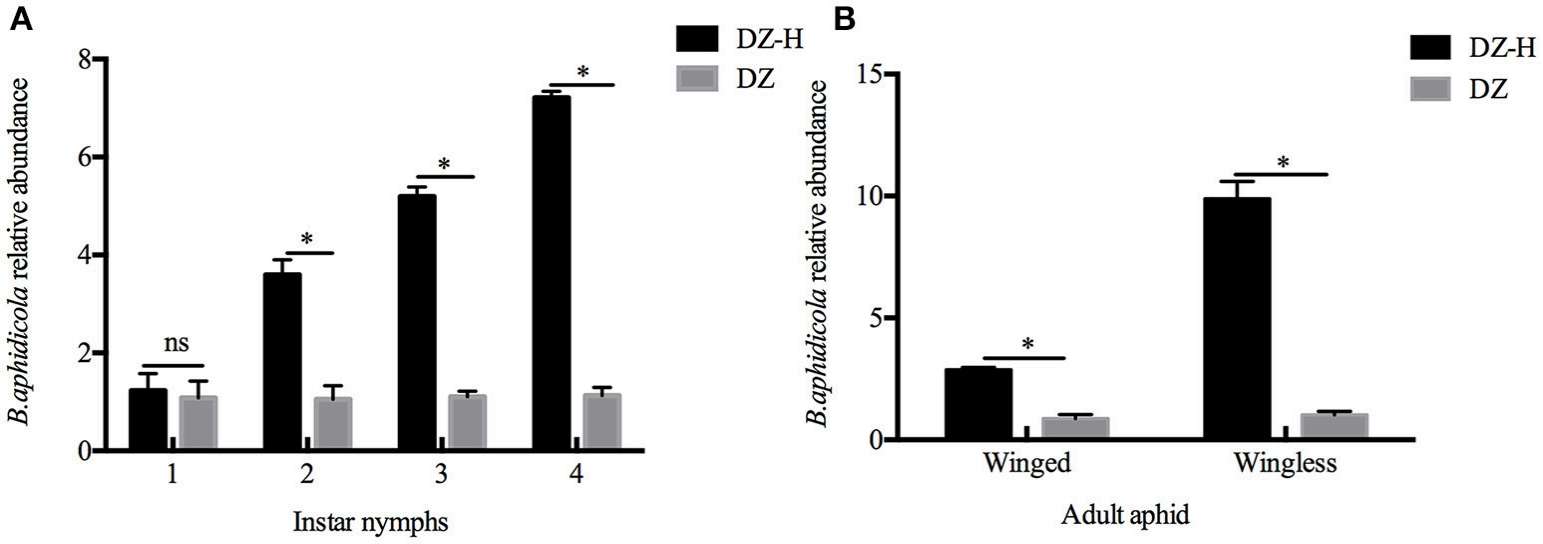
Relative abundance of *B. aphidicola* in the DZ-H and DZ strains. **(A)** Relative abundance of *B. aphidicola* in different instar nymphs. **(B)** Relative abundance of *B. aphidicola* in winged and wingless adult aphids. The asterisk indicates significant differences based on the Mann-Whitney *U*-test for two sample comparison at *P* < 0.05 and ns indicates no significant difference.

The relative abundance of *B. aphidicola* was measured by quantitative PCR. The abundance of *B. aphidicola* in the DZ-H strains was significantly higher than in the DZ strain during the entire development stage, except for the first instar nymphs. The comparison of the abundance ratios of *B. aphidicola* between DZ-H and DZ in different developmental stages of *S. miscanthi* are listed as follows: 1.139-fold (*t* = 0.519*, P* = 0.631), 3.415-fold (*t* = 10.645*, P* < 0.05), 4.712-fold (*t* = 31.191*, P* < 0.05), 6.381-fold (*t* = 50.261*, P* < 0.05) for the first-fourth instar nymph stage, respectively (Figure 4A), and 3.281-fold (*t* = 19.345*, P* < 0.05) and 9.671-fold (*t* = 21.363*, P* < 0.05) in winged and wingless adult aphids, respectively (Figure 4B). These results further confirmed that the relative abundance of *B. aphidicola* was affected by *H. defensa* infection.

## Discussion

Research on the role of S-symbionts in the aphid *S. miscanthi*, one of the most important pests of gramineous crops in China, is still incomplete. In order to obtain target S-symbionts and understand their functions, stable geographical populations with different symbiotic infections are needed. We screened the diversity of S-symbiont infections from established laboratory populations of 17 geographical clones. A remarkably variable composition of S-symbiont infection was evident in *S. miscanthi*. Interestingly, the infection of S-symbionts among the different wheat-producing provinces differed somewhat, but samples from different places in the same province showed a high degree of consistency. It is noteworthy that *H. defensa* has only been detected in Yunnan province with an average elevation of 2,000m, and *S. symbiotic* has only been detected in the Tibet autonomous region with an average elevation of 4,000m. Interestingly, both areas belong to the plateau region of southwest China that features complex geological and topographical conditions. Our geographic sampling data suggest elevation and topography may contribute to the symbiotic diversity. As the mechanism of symbiont distribution in *S. miscanthi* are unknown, however, biotic and/or abiotic factors could produce the regional differentiation, as proposed by Oliver et al. (2014) and Tsuchida et al. (2002). Indeed, other factors that are not mediated by natural selection such as genetic drift, founder effects (Russell et al., 2013) and host plant (Guidolin and Cônsoli, 2017) can also affect the diversity of S-symbionts between geographical populations of *S. miscanthi* in different locations in China. Although we did not investigate the infection rate of symbionts in natural populations, our results, combined with information reported in the literature (Sepúlveda et al., 2016; Zytynska and Weisser, 2016), show that multiple S-symbionts coexist within local populations of *S. miscanthi* in different geographical strains.

*Hamiltonella defensa* is a well-known S-symbiont that confers protection to the aphid against parasitoid wasps (Oliver et al., 2005; Degnan and Moran, 2008; Łukasik et al., 2013; Vorburger and Rouchet, 2016), with a high infection rate of 31.3% (5/16) in US strains (Sandström et al., 2001) and 39.5% (15/38) in UK strains of *A. pisum* (Darby et al., 2001). However, it was not detected (0/119) in Japanese strains of *A. pisum* (Tsuchida et al., 2002; Rothacher et al., 2016). In our study, *H. defensa* was also not widely distributed in *S. miscanthi* from the screened area. It was only present in four clones collected in Yunnan Province. Even though we did not obtain a single *Hamiltonella*-infected *S. miscanthi* strain, two interesting strains (YX and DZ) were collected from Yuxi and Dezhou wheat fields in China. Both strains had the same S-symbiont infection profiles, except for the presence of *H. defensa* (YX: *Hamiltonella*-infected and DZ: *Hamiltonella*-free). By artificial infection, *Hamiltonella*-free strain (DZ) and *Hamiltonella*-re-infected strain (DZ-H) with the same genetic background were generated. After antibiotic treatment, a *Hamiltonella*-decreased strain (DZ-HT) was also obtained.

Fitness measurements revealed that the *Hamiltonella*-re-infected treatment increased the fitness of *S. miscanthi*, as evidenced by greater numbers of offspring, increased weight, and higher wingless aphid rate. Meanwhile, decreasing the abundance of *H. defensa* with antibiotics resulted in lowered adult aphid weight and percentage of wingless aphids. Similar results have been reported for the whitefly (Su et al., 2013), while Oliver et al. (2008) reported that *H. defensa* shortened the mean generation time of *A. pisum*. However, in *S. miscanthi*, research on the influence of *H. defensa* in improving aphid fecundity is sporadic (Łukasik et al., 2013) and other S-symbiont infections were not identified. In our research, we used four fitness indices (number of offspring, weight of adult aphids, percentage of wingless aphids, and longevity) to evaluate the effect of *H. defensa* on the ecological fitness of *S. miscanthi* strains. Surprisingly, the difference in longevity found between DZ and DZ-H strains were not significant, but was only slightly decreased. A close relationship with symbionts can be costly for the host (Polin et al., 2014), with these direct costs caused by a trade-off between allocating resources to symbiosis and other adaptive functions. A previous study found that aphids exhibited many behavioral defenses against enemies and these behaviors had some associated costs, leading to a reduction in aphid reproduction (Dion et al., 2011). We hypothesize that the benefit of *H. defensa* infection in terms of number of offspring, weight, and wingless aphid rate represents a trade-off against the cost of longevity, with an overall benefit of *H. defensa* infection.

*Buchnera aphidicola*, a primary symbiont in almost all aphids, appears to benefit their hosts primarily through the provision of nutrients that are limiting for growth and reproduction (Clark et al., 2000; Oliver et al., 2010). In this study, the relative abundance of *B. aphidicola* in the DZ-H strain was significantly higher than the DZ strain in all but the first developmental stage. This phenomenon implies a linkage of *B. aphidicola* with *H. defensa*, and the difference abundance of *B. aphidicola* in aphid offspring might not be caused by microinjection. The increased in *B. aphidicola* could result from increased nutritional requirements of the whole system. *B. aphidicola* may be “feeding” *H. defensa* since the latter does not seem to be able to manufacture many essential amino acids (Degnan et al., 2009). Therefore, we speculate that infection with *H. defensa* may indirectly improve the fitness of *S. miscanthi* by stimulating the abundance of *B. aphidicola*. In addition, based on the results of the current study and combined with the viewpoint that *H. defensa* inhabits secondary bacteriocytes intercalated between primary bacteriocytes that harbor *B. aphidicola* (Moran et al., 2005b), it can be hypothesized that *B. aphidicola* and *H. defensa* may perform complementary functions in the aphid host and facilitate biological interactions between them. More interestingly, several reports using high-throughput sequencing methods have suggested that *S. symbiotica* and *Wolbachia*, which are facultative symbionts in many aphid species, have evolved to form a deep and co-obligate association with *B. aphidicola* in different aphids (Manzanomarín and Latorre, 2014; De et al., 2015). Although *H. defensa* is not necessary for *S. miscanthi*, it affects the host aphid ecological adaptation to some extent. Further studies employing metagenomics and proteomics techniques will be needed to reveal the underlying mechanism of how *H. defensa* improves the fitness of *S. miscanthi*, as well as to examine the interactions between *H. defensa* and *B. aphidicola* and the interactions among the symbionts themselves.

In summary, we established a feasible method to obtain aphid strains with and without a target symbiont under the same other S-symbiont infection profiles and genetic background by screening different geographical populations and microinjection. In addition, infection with *H. defensa* increased the fitness of *S. miscanthi* and enhanced the relative abundance of *B. aphidicola*. Our results establish a basis for functional studies by revealing the effect of *H. defensa* on host aphid ecology adaptation and the correlation with *B. aphidicola*. A better understanding of the insect and endosymbionts interaction will supply information on the potential value of manipulating microbiota to control insect pests. The diversity and ecological function of symbionts may provide important guidance to field arrangements of wheat varieties in different wheat production areas.

## Author contributions

LQ, FJ, CJ, and WM conceived and designed the experiments. LQ and SJ performed the experiments. LQ analyzed the data. LQ and CJ wrote the paper. All of the authors read and approved the final version of the manuscript.

## Conflict of interest statement

The authors declare that the research was conducted in the absence of any commercial or financial relationships that could be construed as a potential conflict of interest.
